# The Use of a Formative Pedagogy Lens to Enhance and Maintain Virtual Supervisory Relationships: Appreciative Inquiry and Critical Review

**DOI:** 10.2196/26251

**Published:** 2021-10-18

**Authors:** Chloe Louanne Jordan, Thillainathan Sathaananthan, Leo Anthony Celi, Linda Jones, M Abdulhadi Alagha

**Affiliations:** 1 Barts and The London School of Medicine and Dentistry Queen Mary University London United Kingdom; 2 Centre for Medical Education School of Medicine University of Dundee Dundee United Kingdom; 3 Laboratory for Computational Physiology Harvard-MIT Health Sciences and Technology Massachusetts Institute of Technology Cambridge, MA United States; 4 Division of Pulmonary, Critical Care and Sleep Medicine Beth Israel Deaconess Medical Center Boston, MA United States; 5 Department of Biostatistics Harvard T H Chan School of Public Health Boston, MA United States; 6 MSk Lab, Department of Surgery and Cancer Faculty of Medicine Imperial College London London United Kingdom; 7 Institute of Global Health Innovation Department of Surgery and Cancer Faculty of Medicine, Imperial College London London United Kingdom

**Keywords:** medical education, virtual learning, formative pedagogy, supervisory relationships, pedagogy, mentors, education, virtual education, teaching, online platforms, web-based

## Abstract

**Background:**

Virtual supervisory relationships provide an infrastructure for flexible learning, global accessibility, and outreach, connecting individuals worldwide. The surge in web-based educational activities in recent years provides an opportunity to understand the attributes of an effective supervisor-student or mentor-student relationship.

**Objective:**

The aim of this study is to compare the published literature (through a critical review) with our collective experiences (using small-scale appreciative inquiry [AI]) in an effort to structure and identify the dilemmas and opportunities for virtual supervisory and mentoring relationships, both in terms of stakeholder attributes and skills as well as providing instructional recommendations to enhance virtual learning.

**Methods:**

A critical review of the literature was conducted followed by an AI of reflections by the authors. The AI questions were derived from the 4D AI framework.

**Results:**

Despite the multitude of differences between face-to-face and web-based supervision and mentoring, four key dilemmas seem to influence the experiences of stakeholders involved in virtual learning: informal discourses and approachability of mentors; effective virtual communication strategies; authenticity, trust, and work ethics; and sense of self and cultural considerations.

**Conclusions:**

Virtual mentorship or supervision can be as equally rewarding as an in-person relationship. However, its successful implementation requires active acknowledgment of learners’ needs and careful consideration to develop effective and mutually beneficial student-educator relationships.

## Introduction

### Background

For centuries, learning has primarily been undertaken with both students and teachers physically present in a classroom, with the roles and responsibilities of both being fairly well defined. Although distance learning dates back to as early as the 18th century, many supervisors have had little opportunity to reflect on how their face-to-face pedagogical skills can be transferred on the web [1,2]. However, it has arguably gained more popularity since the *virtual explosion of web-based education* following the global COVID-19 crisis [3].

Mentoring and supervision describe two different but overlapping phenomena and are often used interchangeably [4]. Supervisory relationships tend to have a power dynamic parameter and exemplify a formal learning contract wherein deliverables, such as assessments, academic requirements, and program completion, need to be met [5]. Mentorship is seen as a one-to-one relationship, whereby a senior person voluntarily teaches, supports, and encourages another, with the main purpose of sharing knowledge, wisdom, and support [6]. For us, both roles require an element of discipline and support, playing a central role in the students’ overall experience, satisfaction, retention, and research completion.

Despite the multitude of differences between face-to-face and web-based supervision and mentoring, it can be argued that most skills are similar regardless of the environment [7]. Theories, such as the concept of formative pedagogy by Jones [8], which underpins an approach at Dundee University, and *third space*—a component of learning spaces that promote human connections and interactions to cultivate holistic, interculturally enriched experiences—by Elliot et al [9], can provide key foundations for building successful virtual relationships. Both Jones [8] and Elliot et al [9] argue that during disruptive transitions into postgraduate environments, the explicit use of learning contracts generates clear expectations of each participant and can create safe spaces for discussion, support, and problem solving, arising from the tensions among the components of the students’ reality. Formative pedagogy requires the development of reciprocal, trusting, and respectful supervisory relationships through negotiated student-centered *learning contracts*, as promoted by Anderson et al [10].

Transitions in learning are internal, ongoing processes in the mind [11] and happen when moving from one context to another [12,13]. Therefore, when moving from the known, face-to-face to the unknown web-based method, individuals can experience changes in physical, cultural, psychological, and social domains [12,13]. Thus, educators need to facilitate sense making of new rules and routines that operate in learning environments, such as on the web [14]. Benefits of remote learning include learning flexibility for students, more relaxed learning environments, low-cost delivery of courses, and the ability to access resources at a geographical distance from the campus [15-17]. Furthermore, the global accessibility and outreach of remote learning provides an appropriate technical infrastructure to connect academics anywhere to diverse groups of learners [18]. Given the potential pedagogical benefits of virtual learning, it is important to address the barriers that prevent an efficient educator-learner relationship from developing, thereby ensuring that the web-based learning experience is optimized.

In the era of pedagogical innovation, educators may feel under constant pressure to adapt their mentoring or supervisory strategies and embrace enhanced learning technologies that promote stimulating learning environments [19]. This surge in the use of web-based pedagogical activities has led to inquiry and debate into the attributes and skills required in an effective virtual educator. How can educators enhance the educational experience of web-based learners? It is thought that underpinning the role of mentors or supervisors is the capacity to form an appropriate pedagogical relationship, even within the confines of web-based learning. The aim of this study is to compare the published literature, through a critical review, with our collective experiences in an effort to structure and identify the opportunities (what works well) for virtual supervisory and mentoring relationships in terms of educator attributes and skills as well as provide recommendations to enhance virtual learning.

This paper starts by illuminating the relevant educational theories and frameworks of virtual education. It then draws on published literature and a small-scale reflective appreciative inquiry (AI) to understand the contributing factors identified by 5 authors engaged in successful supervisory and mentorship relationships, which have been conducted primarily on the web.

### Educational Theory and Framework

**Figure 1:**
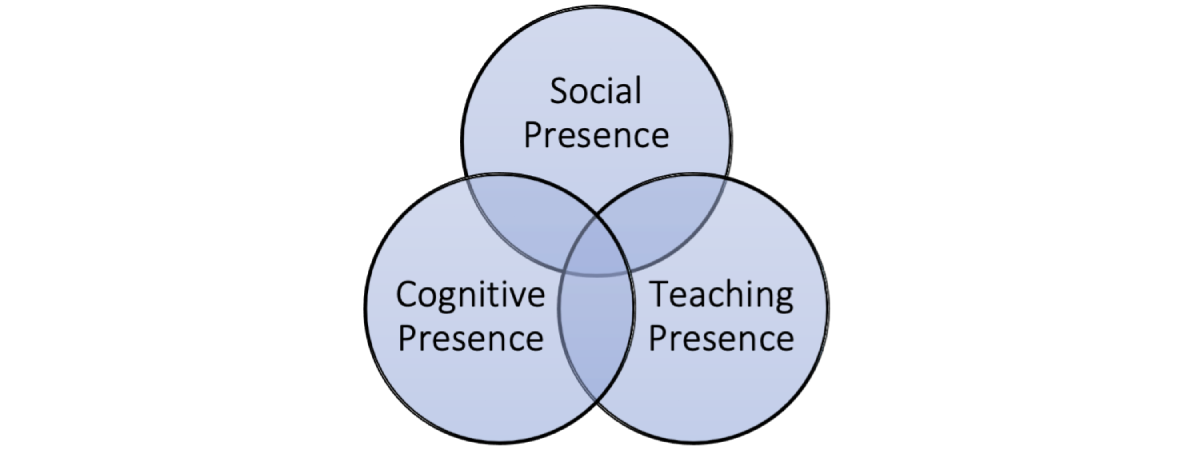
Community of inquiry framework.

No single learning theory has arguably emerged for virtual education [20]. Researchers have recently sought to further develop and formalize models that capture the features of virtual pedagogy [20]. A model that has produced significant interest is the community of inquiry (COI) framework proposed by Garrison et al [21]. They argue that an effective web-based learning experience is best understood based on the concept of three overlapping circles, each representing a distinct *presence*—*social, cognitive,* and *teaching* [21]—as shown in Figure 1*.*

*Social presence* is our ability to establish personal and purposeful relationships encompassing open and effective communication as well as group cohesion [21]. It strives to create an environment for inquiry and quality interaction, including reflection and feedback, to collaboratively achieve educational targets [22]. In other words, it is the quality, not the quantity, of interactions that can lead to progressive discourses. *Cognitive presence* is a process of “exploration, construction, resolution and confirmation of understanding” that occurs through educational partnership and reflective thinking [21].

The final element, *teaching presence,* can be broadly categorized as the *virtual visibility* of the supervisor [23]. In the COI model, teaching presence incorporates the direction, organization, and facilitation of both cognitive and social processes to fulfill personally meaningful and educationally valuable learning outcomes [21]. A number of studies have attested to the importance of teaching presence for a successful virtual learning environment [24-26]. The general consensus in literature is that teaching presence is a significant determinant of “student satisfaction, perceived learning, and sense of community*”* [22].

The COI model conceptualizes effective virtual learning as a result of *interconnectedness* at the heart of learning experiences to deliver high-quality remote teaching [23,27-29]. There is a need to understand the importance of the supervisor-student relationship and how this can be enhanced and developed in a web-based environment. This small-scale study merges evidence-based literature with the authors’ expertise and experience to suggest instructional recommendations that may enhance the effectiveness of distance learning.

## Methods

### Overview

**Figure 2:**
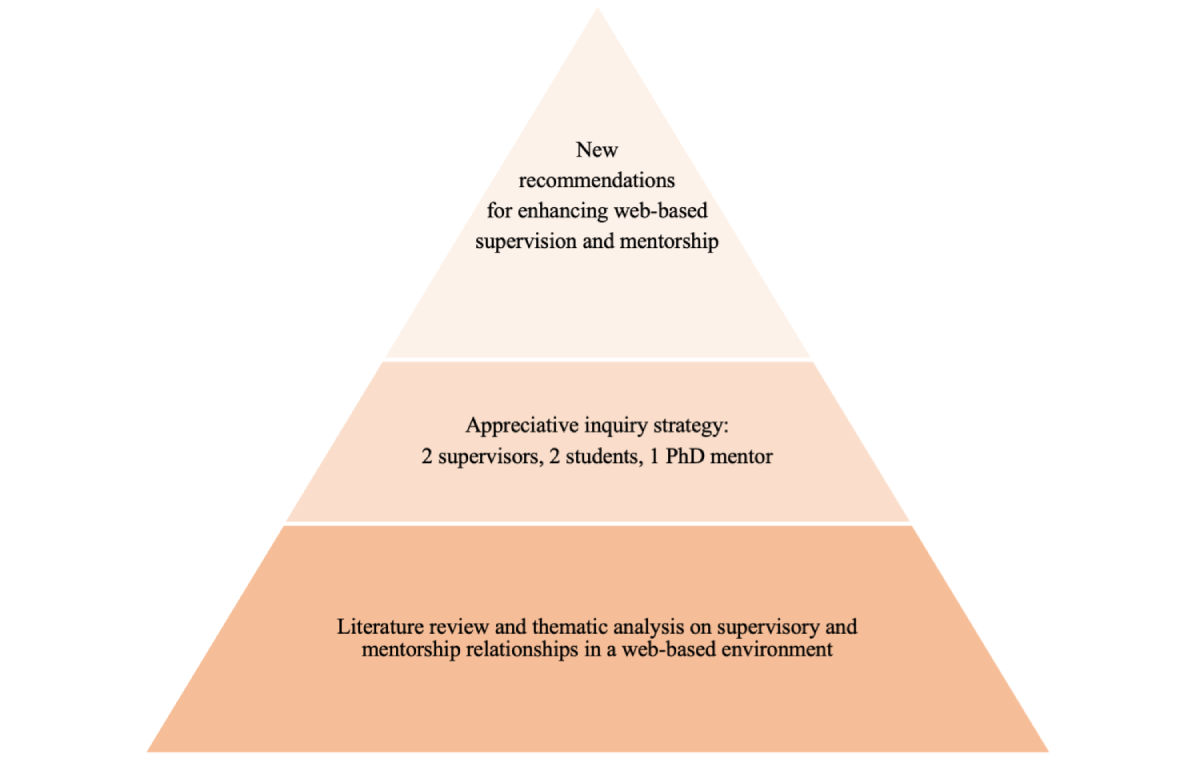
The study methodology pyramid embracing an appreciative inquiry approach.

The methods include a critical review of published literature and thematic analyses of the authors’ views developed through conversation and captured via a qualitative survey, as shown in Figure 2. The survey items, not formally validated, were developed and checked by the authors using the 4D model (discovery, design, dream, and destiny) of Cooperrider and Godwin [30]. The benefits of AI include avoidance of the traditional *deficit-based* paradigm of problem solving and, instead, adopting an *affirmative* approach “to look for what is good in the organization, its success stories” [31].

### Literature Review

A review was performed to capture the published literature on remote supervisory relationships. The search was conducted in PubMed using the following search strategy:

1. Distance OR online OR remote OR virtual
2. AND Supervis*
3. AND relationship* OR guideline* OR strateg* OR tip*
4. NOT technological OR hardware OR software

**Figure 3:**
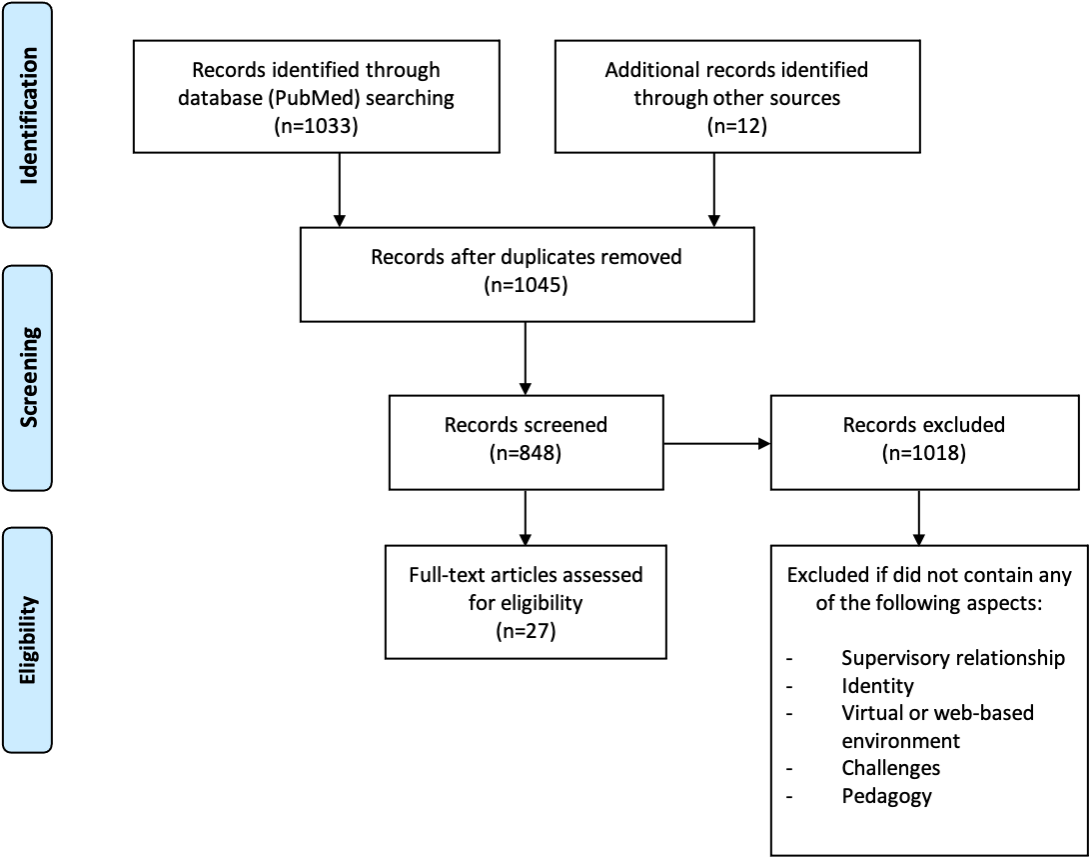
Scoping review of published literature.

Studies were screened for topics related to challenges and barriers in virtual learning and were included if they contained any of the following aspects: supervisory relationship, identity, pedagogy, virtual or web-based environment, and challenges. Figure 3 provides a visual summary of the scoping review.

### Appreciative Inquiry

This study embraces an AI framework, which draws significantly from storytelling [32]. Each author was asked to share their perspectives on five key questions (Textbox 1) regarding what works in their remote supervisory or mentorship relationships. The authors are all interconnected by roles and responsibilities and represent 2 supervisors (of undergraduate, master’s, and PhD), 3 students, and 1 PhD mentor. The supervisory relationships are as follows:

1. Undergraduate: MAA (supervisor) and CJ (student); relationship transitioned from face to face to virtual.
2. Postgraduate masters: LJ (supervisor) and MAA (student); relationship included both face-to-face and virtual elements.
3. PhD: LJ (supervisor) and ST (student); relationship included both face-to-face and virtual elements
4. PhD: LC (mentor) and HA (mentee); relationship has been purely virtual.

**The appreciative inquiry questionnaire.**

- Describe a high-point experience during your remote supervision—a time when you felt most alive and engaged, a moment that captures your supervisory relationship at its best.
- Without being modest, what is it that you most value about yourself and your role and participation in the supervisory relationship?
- What are the core factors that gave or give life to this supervisory relationship without which the quality of web-based supervision would be significantly reduced?
- What three wishes do you have to enhance learning opportunities from web-based supervision?
- On the basis of what worked for you, what advice would you offer to other supervisory dyads?

According to Richards [32], AI is a philosophy that aims to determine an organization’s fundamental strengths instead of focusing on overcoming problems and then maximizes and builds on those aspects. This approach results in a greater holistic, unified, and successful process of change. Figure 2 illustrates the AI methodology used in this study.

It is argued that a large part of a successful supervisor-student relationship is deeply rooted in the human interaction between the participants in the relationship [33,34]. Thus, supervisory relationships in a virtual environment were explored using the personal accounts of the authors' experiences in web-based supervisory relationships. The participants explored their responses during a video-call, and recorded, transcribed and thematically analyzed them.

## Results

**Figure 4:**
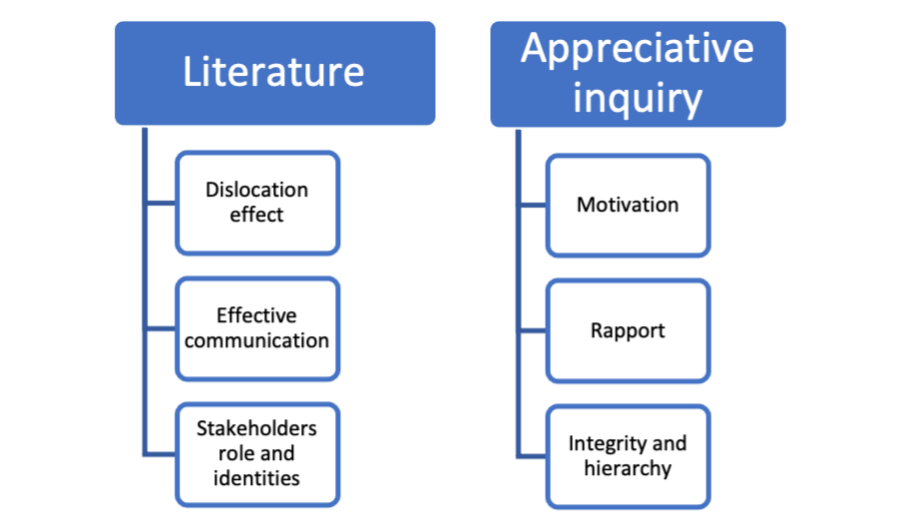
Summary of thematic analysis from literature review and appreciative inquiry.

**Figure 5:**
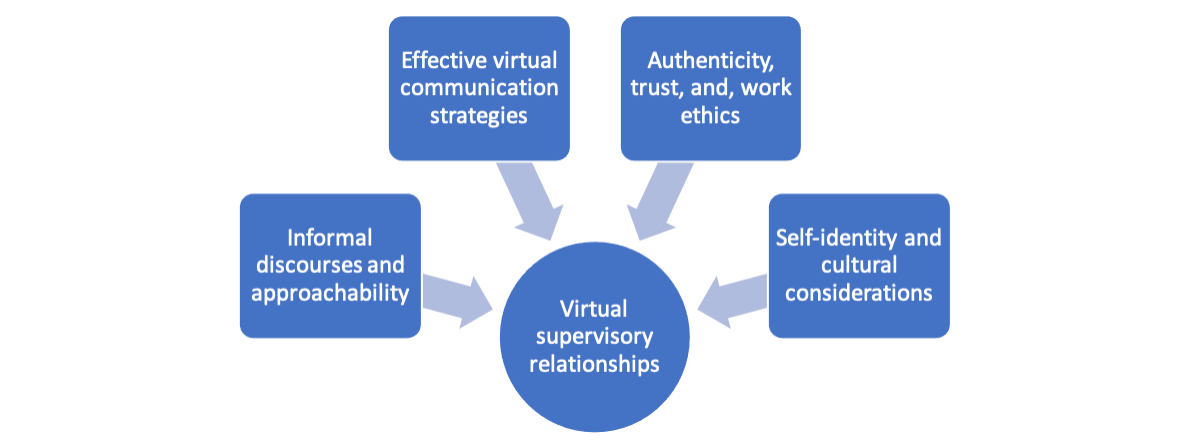
The 4 key dilemmas related to virtual supervisory relationships.

The findings summarized below highlight the themes in the literature and from the AI approach (Figure 4). The major themes identified in the literature on virtual supervisory relationships were *overcoming the dislocation effect,* encompassing *effective communication strategies, and negotiating stakeholder roles and identities.* The richly descriptive themes that emerged from AI narratives linked closely to those identified in the literature and include motivation, rapport, integrity, and hierarchy. Figure 5 highlights the four key dilemmas related to virtual supervisory relationships as demonstrated in the author’s AI.

## Discussion

### Informal Discourses and Approachability to Overcome the Dislocation Effect

Nasiri et al [35] found that many of the challenges in web-based supervision arise from the spatial and temporal distance between supervisors and students. From a timing viewpoint, issues may arise in finding a mutually convenient meeting time for both parties to connect. Web-based learning involves experiences of dislocation [36]. As a result of this *dislocation effect*, students may tend to feel isolated, disorientated, and perhaps disengaged, all of which are barriers to forming a good supervisory relationship [37]. These feelings tend to drive the supervisory conversations towards a more formal format, with both parties lacking in personal knowledge about each other, making it more difficult to create an environment for informal discourse [35].

Previous studies have suggested that occasional informal social web-based interactions can contribute to effective virtual supervisory relationships [38]. The importance of informal supervisory relationships was echoed by all authors in the group AI, which suggested that learners may benefit from a combination of group and individual web-based meetings to help students feel engaged, encourage collaborative learning, and build peer relationships. This suggestion aims to cultivate a sense of belonging and community and represents a type of *social presence*, as seen in the COI framework. The authors suggest that sharing progress, experiences, and challenges with peers aims to combat feelings of isolation and disconnectedness. The AI framework further revealed that the extent to which the supervisor was perceived to be available to the student influenced the supervisory relationship. The authors agreed that supervisors who were perceived to have an approachable *web-based presence* seemed to have a more positive relationship with their students and helped in breaking down the dislocation effect:

Virtual platforms if anything helped me develop a stronger bond with my mentor, who is always accessible with a quick response. Our relationship dynamic shifted with time to more informal, and I felt that there was nothing that I couldn’t discuss with my mentor. This helped us not only in building trust and confidence in our relationship but also we came up with new ideas—that’s always an eureka moment.

Views of MAA and LC, echoed by other authors

One PhD dyad adapted the five-part temperature reading process of Satir [39] to “build a connection and learn to communicate on important topics,” to facilitate congruent communication, cultivate meaningful relationships and to use as a conflict resolution tool if needed. The framework is based upon the supervisor and student taking turns to share information from five domains: appreciations, new information, puzzles, worries and concerns with recommendations for change, and hopes and wishes.

The PhD student found the following about this system:

it provided room to talk and openly share feelings, the structure generated dialogue and a sense of real and equal participation new to someone from a more hierarchical system.

View of ST

This combination of Satir’s communication tool [39] with a shared commitment to Nodding’s [40] 3R principles of reciprocity, relatedness, and responsiveness, and abiding to where “carer and cared for contribute appropriately” [41] to any student-teacher relationship and are well aligned with the concept of formative pedagogy developed by Jones [8], enhancing trust in and take up of formative assessment by the learner:

I worked with my supervisor face-to-face prior to the pandemic and we both worked on building an informal culture of mutual trust and interest. What enhanced this relationship further was knowing that my supervisor cared about my well-being through occasional informal calls once lockdown measures were in place [...] I felt valued as a student and knew that they were there for me to develop personally and professionally.

View of CJ

Specific examples that facilitated the development of good supervisory relationships included a preagreed framework for authentic checking in and catching up. One of the authors described the following:

When most countries went into lockdown, my supervisor took the initiative to organise daily group virtual workout sessions with his team across the globe. I believe this was an opportunity to check in on each other and allowed me to strengthen my sense-of-belongingness to my supervisor and his research family [...] I had first-hand experience of him genuinely caring about everyone in his team in a supportive and friendly environment.

View of MAA

In comparison with the author’s findings, a number of studies from the literature search highlighted that differences in technological literacy between students and supervisors exacerbated the dislocation effect by disrupting the exchange of information, thereby reducing the desire for interaction [42-45]. This was not highlighted as an issue for the authors, whereas the AI framework highlighted that these differences aided in breaking down formal barriers by encouraging greater informal discourse, thereby building a greater rapport. Furthermore, the studies that highlighted an increase in dislocation effect investigated students and supervisors who were located in different countries with imbalanced resources, which was not the case for the supervisory relationships in this study [1,37]. Therefore, although the authors of this paper experienced differences in technological skills in their supervisory relationships, the main differences in a large number of published articles were in the availability of resources and infrastructure [1,7]. A challenge that was not heavily experienced by the authors but was predominately referenced in the literature related to time zone differences contributing to problematic synchronous communication [46].

### Effective Virtual Communication Strategies

Virtual environments provide ease of communication and global outreach [1]. With the advent of web-based technologies, it is now possible to be mentored or supervised by any chosen individual worldwide. It may also minimize time, cost, hierarchy, and stress related to commuting or being physically present in an unneutral workplace environment [47]. However, studies have shown that, unlike in classroom-based teaching, distance students are limited in the quantity of interaction they have with their supervisor, thus limiting the amount of guidance and feedback the student receives [42,48].

However, the perceived drawbacks of virtual learning are arguably issues with in-person learning as well [42,48,49]. Communication difficulties are thought to be individual-specific, and the same people who have difficulties with in-person mentoring may experience challenges with virtual mentoring.

All authors highlighted the importance of relationship building and trustworthiness in supervisory relationships, whether in person or virtual:

I am genuinely committed to creating a relationship with my students and build trust within the confinements of the relationship. I try to convey that I am a trustworthy person, and my aim is to help them succeed. The relationship is an opportunity for meeting of minds; I may be different to my students, but it doesn’t mean I’m better than them, I occupy a hierarchical position in terms of the task but not as a human being.

LJ view

Communication of feedback has been highlighted as a difficulty in a web-based environment [50]. It can be argued that with fewer interactions, it is harder to maintain quality feedback. For instance, with the accessibility of services such as the tracking facility of Microsoft Word, there is a tendency for students to accept additions and amendments from the supervisor, thereby eliminating potential reflection and constructive discussion [42]. This method of feedback does not promote the motivation, engagement, and independence of the student and can lead to an overreliance on the supervisor. The limitations of verbal or nonverbal cues from the student may reduce the opportunities for supervisors to check the students’ understanding of feedback and risk a more hierarchical system of feedback in comparison to a mutual, bidirectional learning experience [35,49].

As a result, giving and receiving quality feedback can be challenging and require an empathetic and reassuring skill set to achieve [42]. It is important for faculty members to learn how to give web-based feedback and understand the nature of their students’ emotional and academic needs [49]:

Most faculty are not taught how to conduct online relationships, give online feedback, or how to compensate for the lack of body language cues. If I want to feel sad, then I have to show you that I’m feeling sad. There is a real need for faculty development to shift towards the space between us, help people understand the nature of feedback, to internalize things and avoid future errors.

View of LJ, echoed by other authors

When communicating, it is important for supervisors to be aware that feedback has emotional connotations, and thus, it should be structured in an appropriate manner [50,51]. Feedback is affective, and the literature [52] suggests making use of Hyatt’s [53] *phatic* comments, where the aim is to create and maintain a good social and academic relationship between the supervisor and the student [53]. This type of comment is used to express praise, register interest, or encourage, for example, “This is a well presented and well written assignment.” Similarly, Hyland and Hyland [54] documented ways in which educators can mitigate their criticism. This strategy is reflected by using hedges such as *might*, *possibly*, and *maybe* or asking questions and suggesting points for reflection such as “have a think about [...]”, as opposed to direct comments [54].

The collective experience from this AI survey highlighted that virtual meetings work best when characterized by negotiated agendas, when a safe environment is created, and where disagreements are looked for and can be constructive:

Once a student enters my academic bubble, I make it clear to them that we both have equal power dynamics; we both need to agree on agendas and next steps, and I welcome feedback from my learners. Just like the student, I love a challenge and a disagreement—this is how we can come up with innovative ideas and reflections.

View of MAA

I value having an agenda prior to meetings while maintaining an open relationship with my supervisor. I see it as a system of appreciation, puzzles and criticisms with recommendations based on behaviours and if there is an issue it will naturally come up [...]

View of ST

The literature proposes, as agreed by the authors [55,56], that the main priority in a new supervisory relationship is establishing communication. A proposed method for achieving this is by developing a communication strategy that includes who, when, and how [57]:

1. *Who*: Watt [58] suggested that “maintaining effective communication is the responsibility of the supervisor.”
2. *When*: A balance needs to be struck between student independence and an overreliance on the supervisor. It has been highlighted in the literature that regular meetings reduce the potential isolation of students and encourage them to be motivated and feel supported in a web-based environment [49]. Studies have shown that a student’s progress and satisfaction with web-based learning are influenced by the frequency of virtual meetings [59-61].
3. *How*: Research has shown that supervisors and students should choose technologies based on their familiarity and how appropriate they are to the specific meeting goal [62,63].

The consensus from the authors is to encourage an approach where supervisors *ask questions rather than tell* and *have a conversation* when providing feedback. It is recommended to allocate time to scaffold the student to *feed forward*; this not only allows the student to structure their next steps but also agree to a plan so that the advice and suggestions can be acted upon.

### Authenticity, Trust, and Work Ethics

Trust is a key factor in social relationships and has been described as an important determinant of achievement within organizations [64]. Numerous definitions of trust have been put forward with the commonalities referring to expectations, beliefs, or attitudes towards the other person and the willingness to trust, in addition to the degree of vulnerability that results from the risk of trusting another person. Trust forms an integral part of building successful academic relationships, and it grows when supervisors and students allow themselves to be vulnerable and when tensions arise between the two parties [56]. As opposed to seeing these challenges as issues, they may be seen as opportunities for trust to grow. Arguably, trust and relationship building in virtual settings require a different framework and may be better positioned than their traditional counterparts [65]. A few studies have highlighted that trust becomes ever more important in virtual environments to minimize the psychological distance among team members and to create unity [66,67].

According to prior work [68,69], trust is the core variable and one of the most influential factors for all aspects of team work and success. Trust has a significant effect on performance [70] and can be considered as the binding unit that facilitates collaboration [71]; it is of particular importance in web-based education, where interactions may lack contextual and nonverbal cues [72,73]. The literature search highlighted that trust results in greater team collaboration, including commitment, motivation, and communication [74]; however, it is more difficult to establish and maintain trust virtually [75,76]. Maurping and Agarwal [77] highlighted that early trust building in virtual relations is crucial to developing a functional working relationship. When early trust is established, team members gain confidence to participate in behaviors and actions that improve team performance [71].

The results from the authors’ AI are supported by published literature that reported that adopting a social approach when working in a virtual environment, such as encouraging social discourse early in the collaboration [66] or creating opportunities and time for informal, casual and non–work-related interactions [75], can all improve trust. One study investigated the challenges associated with trust in a web-based environment and found that the absence of nonverbal cues, such as body language, reduced tone of voice and inflections, along with a lack of facial expressions and difficulties inferring the intentions of others, delayed the participant’s decision to trust a new team member or not and reduced the expression of their own trustworthiness. The results of this study are reinforced by Olson and Olson [75] who state that the use of a webcam during communication aids in instances where team members do not know each other.

One trait identified from the AI survey is that supervisors and students valued authenticity and the ability to be their *true-self* within a supervisory relationship. It highlighted the belief that being able to authentically engage with one another creates an environment conducive to building trust and personal connections. The authors recommend that in order to establish trust at the beginning of the supervisory relationship, an introductory ice breaker or a trust-building exercise can be used to increase student participation, self-esteem and also nurture and foster the supervisory relationship:

For me, the core factors that give life to this supervisory relationship are trust, knowing that this person cares appropriately, capacity to solve problems and authenticity (true-self). Trust encompasses personal, emotional and practical trustworthiness and if someone says they will do something, they will [...] Appropriate level of challenge both ways as this is where critical thinking emerges-depends on clear trust.

View of LJ, agreed by all authors

In our experiences, for trust to grow, the supervisor may need to acknowledge the student’s individual needs and circumstances and offer guidance. Relationships in which the student feels truly valued and their supervisor has their best interests at heart may lead to more trusting relationships. Work ethics and maintaining a shared goal between the mentor and the learner, as well as a willingness to be open and embrace different ideas and cultural strategies, may all be successful ways to establish trust:

Shared interest in the project and in an output that will contribute to the knowledge base is a quality without which online supervision may be significantly reduced.

View of LC

Measuring trust quantitatively is difficult because of the complexity of the construct. The literature on building trust within virtual environments is particularly focused on trust within a team setting as opposed to individual relationships [78,79], which was the focus of our AI; hence, it may be difficult to compare apples with pears. However, similarities were identified, including a study by Marlow et al [80], who reported that the development of trust is improved by initial face-to-face contact at the start of the relationship. In addition, the concept of *true-self* that arose within the AI framework has been echoed in other studies exploring the development of trust in web-based environments of which personal traits and characteristics of team members were identified to play a role in establishing trust. These characteristics included ability, integrity, competence, fairness, honesty, and openness in addition to each individual having a level of autonomy [69].

### Sense of Self, Self-identity, and Cultural Differences

A further, more complex theme highlighted is *identity*. Personal variables tend to be crucial in the supervisor-supervisee dyad: age, gender, personality, ethnicity, and culture can all pose challenges and have implications on supervisor-student interactions [42]. The learners’ sense of self may not be a fixed entity but undergoes a process of continuous transformation during their educational experience [81,82]. This construction and deconstruction of self through a continuous process of interacting with self and educational communities may buffer the self-creativity of graduates and harness their resilience and academic success [83,84]. Educators may benefit from an awareness of the learners’ self-identities:

I think it is quite vital to recognise learners’ prior experiences and different personal and professional identities. One of my students, though a medical student, took a year to undertake an intercalated science degree. Recognising and valuing their identities helped me guide their talent and creativity

View of MAA

This is in line with studies by Costello [85] and Monrouxe [86], who conceptualized professional identity formation for health care professionals to be as important as skill and knowledge acquisition and advised integrating graduates into various social settings to optimize their sense of self [85,86].

Linked with the theme of identity is *role ambiguity*, identified as a learning barrier in a virtual learning environment [87]. This phenomenon highlights inconsistency and lack of clarity between student and supervisor expectations in a virtual relationship. This ambiguity can lead to disengagement and unsociability because of the lack of an agreed agenda, expectations, and standards on role behaviors and functions [56,88]. Methods that worked for the group were defining and developing a mutual understanding of individual roles and responsibilities within the supervisory relationship, which could be formalized via a learning agreement or contract.

The AI highlighted that the authors concur with the consensus in literature regarding the configuration of the traditional definition of canonical knowledge, the power and expertise of the teacher, and the passivity and role of the students that has resulted from the virtual environment [89]. Despite the AI framework highlighting the challenges faced by students as a result of the dislocation effect, it failed to address the challenges faced by supervisors. In the literature, the dislocation effect and self-identity have further been described from the perspective of the supervisor and not solely the student. It has been argued that because of virtual learning, the role of the educator has shifted from “gods of knowledge to directors of or leaders in the pursuit of knowledge,” which has the potential to result in professional or self-disorientation [43,90]. Educators may experience a sense of dislocation and a loss of self-identity as their role has changed from the traditional perception regarding their authority, subject knowledge, and expertise. A potential reason as to why this challenge was not experienced by the authors could be that all the supervisors in the group were accustomed to adopting an open and egalitarian approach to the supervisory relationships, as evidenced in the quote below. In addition, in this instance, all supervisors had previous experience with virtual supervision before the pandemic whereas a large proportion of the reported studies emerging as a result of COVID-19, both supervisors and students were novices to the web-based environment:

Given my extensive experience in virtual pedagogy, I believe that formalising a learning contract where the student plays an active partner role is important to enhance their online learning opportunities [...] I would like the student to share who they are, what they need from me and what they want from me.

View of LJ

Remote supervision tends to bring together parties from different geographical regions and cross-cultures; thus, there is a growing realization that cultural differences and intercultural communication are important factors in supervisory relationships. The literature has highlighted that social and cultural differences may influence interactions between students and supervisors [35]. From the authors’ experiences, it was evident that in some South Asian cultures, students are more reserved and less likely to proactively communicate their emotions, opinions, or views because of interpersonal politeness; in some instances, this led to miscommunication and conflict. The findings from the AI framework are supported by the work of Venter [91], who conducted a distance-learning study investigating the role of culture in students. Venter [91] highlighted that there are differences in attitudes regarding authority, which showed varying expectations of the student and supervisor role. The study concluded that differences in expectations arise between students from cultures that view the supervisor-student relationship through a collectivist model (supervisor-centered approach) and those from other cultures who uptake an individualistic model (student approach) [91].

In one student-supervisor relationship, the student (ST) was more accustomed to a teacher-centered approach and initially found it difficult to engage in conversation and discuss their opinions with the supervisor as this was not culturally acceptable for them. Therefore, it required an open discussion regarding their roles in the relationship and management of expectations. Although it has been suggested that students who adopted a collectivistic model of learning experienced greater isolation [92,93], this was not the case in this study. A potential reason for the differences between our study and literature is that most studies in the literature that assessed the role of culture involved undergraduate learners; however, ST is a doctoral student who has been accustomed to working individually in previous degrees and is a senior lecturer in his native country, Sri Lanka. Furthermore, language barriers have been reported to contribute to students’ isolation [94]; however, this cannot be implied in this study, as this was not a barrier in any of the authors’ relationships. This experience of adopting a collectivist model was only experienced by one author and, therefore, may not be representative of other students. However, group AI revealed how their supervisor-student interactions were experienced as dynamic, engaging, and reflective while embracing cultural and background differences.

In my hierarchical culture, Tamil, students are expected to be passive. When I was invited to express my opinion by my supervisor, it was the first time a teacher asked me to do this. I felt that was the best moment of my educational journey and motivated me to be more interactive in the supervision.

View of ST

Both parties in the relationship may find it helpful to have an awareness of the other member’s cultural norms and any differences that may potentially cause conflict. The authors recommend that both parties adopt an inquisitive nature and an acknowledgment that learning does not take place in people’s heads alone; it takes place in people’s hearts and in their lives.

### Limitations and Future Work

There are a few limitations to this study. Although the AI study design was not aimed at generalizing our findings to other educational contexts, this study triangulated the experiences of students, mentors, and supervisors at 3 different institutions in the United Kingdom and the United States. We hope our approach is better assessed by what it conveys in terms of plot, participants, and place while convincing readers of its representativeness. Our small-scale questionnaire, although not validated, was designed to highlight themes and provide preliminary data for more inclusive research in the future.

Further larger scale research using a validated questionnaire across a greater number of institutions is needed to demonstrate whether themes identified are common among supervisory relationships and present in those beyond these authors and their respective institutions. In addition, future studies could usefully address disparities in access to technology, the influence this is likely to have on supervisory relations, and ways in which digital inequalities can be addressed.

### Conclusions

Drawing on published literature and a small-scale AI, our study identified key dilemmas that enable us to perceive our virtual supervisory and mentoring relationships as effective and beneficial. Virtual environments can be as rewarding as in-person relationships and provide innovative opportunities, including global outreach and flexibility, ease of communication, and the potential ability to reduce time, cost, hierarchy, and stress related to physical presence in the workplace.

Our findings propose suggestions to enhance web-based learning experiences, which actively acknowledges learners’ needs, especially in areas related to effective communication, cultural differences, self-identity recognition, and trust building. Careful consideration of these key dilemmas, all of which can act as barriers to an effective supervisory relationship, should be encouraged and recognized for the successful development of effective and mutually beneficial virtual student-educator relationships. However, future inclusive research on ways to manage and address these key dilemmas of virtual pedagogical relationships is needed.

The rapid proliferation of distance learning poses an excellent opportunity for institutions to invest in developmental activities that not only inform but also engage and prepare both students and supervisors for the web-based environment. By investing in formative web-based pedagogy and faculty development initiatives, institutions can empower both learners and faculty to reach their full potential [95].

## Abbreviations

AI: appreciative inquiry
COI: community of inquiry
